# A Remote Sensing Image Super-Resolution Reconstruction Model Combining Multiple Attention Mechanisms

**DOI:** 10.3390/s24144492

**Published:** 2024-07-11

**Authors:** Yamei Xu, Tianbao Guo, Chanfei Wang

**Affiliations:** School of Computer and Communication, Lanzhou University of Technology, Lanzhou 730050, China; 17693164650@163.com (T.G.); wangchanfei@163.com (C.W.)

**Keywords:** remote sensing images, super-resolution reconstruction, window-based multi-head self-attention, convolutional attention, long-range dependencies

## Abstract

Remote sensing images are characterized by high complexity, significant scale variations, and abundant details, which present challenges for existing deep learning-based super-resolution reconstruction methods. These algorithms often exhibit limited convolutional receptive fields and thus struggle to establish global contextual information, which can lead to an inadequate utilization of both global and local details and limited generalization capabilities. To address these issues, this study introduces a novel multi-branch residual hybrid attention block (MBRHAB). This innovative approach is part of a proposed super-resolution reconstruction model for remote sensing data, which incorporates various attention mechanisms to enhance performance. First, the model employs window-based multi-head self-attention to model long-range dependencies in images. A multi-branch convolution module (MBCM) is then constructed to enhance the convolutional receptive field for improved representation of global information. Convolutional attention is subsequently combined across channels and spatial dimensions to strengthen associations between different features and areas containing crucial details, thereby augmenting local semantic information. Finally, the model adopts a parallel design to enhance computational efficiency. Generalization performance was assessed using a cross-dataset approach involving two training datasets (NWPU-RESISC45 and PatternNet) and a third test dataset (UCMerced-LandUse). Experimental results confirmed that the proposed method surpassed the existing super-resolution algorithms, including Bicubic interpolation, SRCNN, ESRGAN, Real-ESRGAN, IRN, and DSSR in the metrics of PSNR and SSIM across various magnifications scales.

## 1. Introduction

As a result of rapid advancements in remote sensing technology, high-resolution remote sensing images have become a crucial geographic information source for various applications, including environmental monitoring, crop type identification, land use, disaster management, weather forecasting, geographic object detection, and biophysical parameter estimation [1,2]. However, due to the physical limitations of remote sensing hardware, imaging systems are susceptible to platform vibrations, optical diffraction, and interference from system noise. These factors often result in blurred images with limited information, which substantially restricts the application scope and measurement accuracy of remote sensing data. In addition, hardware improvements are often cost-prohibitive and provide only limited benefits. To address these issues, software-based image super-resolution techniques, particularly methods driven by deep learning, have been designed to overcome physical constraints and offer an effective means of enhancing remote sensing image resolution.

Super resolution (SR) involves reconstructing a low-resolution (LR) input image into a high-resolution (HR) output image, which is not only more visually appealing but also contains more information [3,4,5]. Recently, the application of deep learning-based image SR techniques has led to significant advances, some of which are tailored specifically for remote sensing. In 2016, Liebel et al. [6] first applied a super-resolution convolutional neural network (SRCNN) [7], based on a standard CNN, to the reconstruction of single remote sensing images. In 2017, Lei et al. [8] proposed a local–global combined network (LGCNet), based on image super-resolution reconstruction, to address the challenges posed by large-scale variations in remote sensing images. In 2020, Xie et al. [9] proposed an SR reconstruction network for remote sensing data, termed an improved residual network (IRN). This algorithm was based on a 32-layer residual CNN and effectively cascaded feature information across different layers, harnessed additional contextual information, established more comprehensive mapping relationships, and enhanced propagation by reusing features. Although CNN-based SR reconstruction methods have successfully been applied to remote sensing images, certain limitations remain. These restrictions are often the result of using convolutional layers with a local receptive field, which affects the processing of global information and long-range dependencies. However, such capabilities are critical for understanding and reconstructing large-scale image features.

Generative adversarial networks (GANs) [10] have been widely used in computer vision tasks, including GAN-based image SR reconstruction methods. In 2018, Wang et al. [11] proposed an enhanced super-resolution generative adversarial network (ESRGAN), which improved SR reconstruction by adding residual dense blocks in the generator and removing optimization steps such as batch normalization. Wang et al. [12] later continued to optimize the ESRGAN architecture and proposed training a real-world blind super-resolution model using pure synthetic data, producing a real-world image ESRGAN (Real-ESRGAN). Owing to the high complexity and diversity of remote sensing data, applying GANs to the SR reconstruction of remote sensing images can introduce unrealistic details or textures [13,14,15]. This issue is particularly pronounced in highly detailed regions, such as urban environments and natural landforms. These unrealistic details can compromise image value in applications such as land cover classification and change detection.

The success of a transformer model [16] in natural language processing has motivated the use of multi-head self-attention (MSA) for computer vision tasks. The primary advantage of MSA is its ability to process long-range dependencies in sequential data. When applied to image processing, transformer-based models can assess the relationships among all pixels in an image via MSA, thereby capturing complex global dependencies. Specifically, the Swin-Transformer [17] architecture addresses excessive elements and computational overhead in traditional transformer attention calculations using window-based MSA. However, this focus on global processing can result in the overlooking of local textures and details, such as subtle changes in vegetation, small bodies of water, or man-made objects in remote sensing images. These elements can be critical for applications such as environmental monitoring and resource management.

Additionally, researchers have applied convolutional attention to construct effective image SR reconstruction networks. In 2018, Zhang et al. [18] proposed the residual channel attention network (RCAN), which enhances a model’s focus on the local importance of an image. By incorporating channel attention (CA), the model dynamically recalibrates channel feature responses, thereby enhancing useful features and suppressing irrelevant information. In 2020, Dong et al. [19] proposed the dense-sampling super-resolution network (DSSR) for the SR reconstruction of large-scale remote sensing images. This framework included an extensive feature attention block that utilized the effectiveness of CA to enhance network representation capabilities. In 2021, Wang et al. [20] applied CA and spatial attention (SA) to a deep dense residual network to enhance the effectiveness of SR reconstruction. However, because CA and SA are both convolution-based attention mechanisms, their effectiveness is still constrained by the size of the convolutional receptive field.

In summary, this study introduces a new SR reconstruction model for remote sensing images, which combines MSA with CA and SA mechanisms. This synergy enables the model to better understand and reconstruct complex scenes, and thus improves reconstruction accuracy. The foundation of this model is a multi-branch residual hybrid attention block (MBRHAB), used for deep feature extraction. First, the MBRHAB extracts global feature information from remote sensing images by utilizing the long-range dependent modeling capabilities of windowed MSA, and then integrates extracted global feature information using a multi-branch convolutional module (MBCM). This MSA mechanism can dynamically adjust attention to various geographic regions depending on task requirements. For instance, when processing images of urban areas, attention can be dynamically assigned depending on the density of buildings, the distribution of roads, or vegetation cover. This capability allows the model to maintain robustness to features in varying seasons and weather conditions. Second, a convolutional block attention module (CBAM) [21] is introduced, which includes both CA and SA, enabling the model to accurately recognize and extract locally important detailed feature information from remote sensing data. This convolutional attention component complements the lack of locally important details during feature extraction. Its combination with MSA more fully utilizes the advantages of global and local features. Finally, the parallel combination of multi-head and convolutional attention modules reduces the computational burden.

## 2. Model Architecture

**Figure 1:**
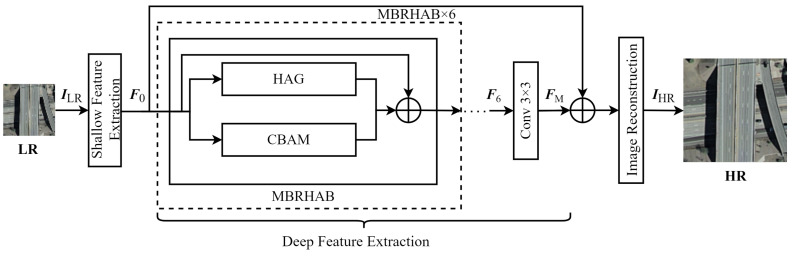
A general overview of the model architecture.

The proposed SR reconstruction model combines multiple attention mechanisms to utilize the characteristics of remote sensing images exhibiting high complexity, large-scale variations, and rich local detail. The model architecture consists of three primary components: shallow feature extraction, deep feature extraction, and image reconstruction, as shown in Figure 1. In this process, LR remote sensing images are first passed through a shallow feature extraction stage, in which superficial image features are collected. A deep feature extraction stage subsequently retrieves more complex feature information. The model then employs residual skip connections to merge image features from the first two stages, and the final stage of reconstruction rebuilds the samples into HR remote sensing images.

### 2.1. Shallow Feature Extraction

Shallow feature extraction was used to capture superficial features in LR remote sensing images, focusing primarily on basic structural information such as edges and textures. These basic features are typically simple to extract, thus requiring fewer convolutional layers and reducing algorithm complexity. As such, this model utilized a convolutional layer with a kernel size of 3×3 to construct the shallow feature extraction module, which adjusted the number of channels from 3 to 64 in the processed images. Shallow feature extraction was implemented using the following formula:

(1) $$F_{0}=C_{\mathrm{SF}}\left(I_{\mathrm{LR}}\right)$$

where $C_{\mathrm{SF}}\left(\cdot \right)$ denotes a convolution operation, $I_{\mathrm{LR}}$ represents an input LR image, and $F_{0}$ is the output from the shallow feature extraction stage.

### 2.2. Deep Feature Extraction

The deep feature extraction stage is primarily intended to capture complex information from images with large variations in target feature scales. The proposed stage includes a novel MBRHAB module, which combines window-based MSA with CA and SA mechanisms. Feature maps were processed using both global long-range dependency modeling and local important feature extraction, facilitating the reuse of features through residual skip connections. A parallel architecture was then used to combine global and local information, thereby enhancing model representation capabilities. The deep feature extraction stage utilizes six MBRHABs, an empirical quantity determined from experiments. The upper branch in each MBRHAB is comprised of a hybrid attention group (HAG), while the lower branch integrates channel and spatial attention in a convolutional block attention module (CBAM).

#### 2.2.1. The Multi-Branch Residual Hybrid Attention Block (MBRHAB)

As shown in Figure 1, the upper branch of the MBRHAB is an HAG, used to extract global features, while the lower branch is a CBAM, which focuses on local features. The HAG and CBAM modules were combined using parallel connections to establish the MBRHAB. This parallel two-branch structure allows for simultaneous feature extraction from both branches, significantly enhancing model efficiency. The MBRHAB extraction of deep features can be expressed as follows:

(2) $$F_{i}=f_{HAG}\left(F_{i-1}\right)+f_{CBAM}\left(F_{i-1}\right)+F_{i-1},\;i=1,2,\cdots ,6$$

(3) $$F_{M}=C_{3\times 3}\left(F_{6}\right)$$

where $F_{i-1}$ represents feature information output from the previous MBRHAB, $f_{CBAM}\left(\cdot \right)$ denotes the CBAM feature extraction operation, $f_{HAG}\left(\cdot \right)$ signifies the HAG feature extraction step, and $F_{i}$ is the output of the ith MBRHAB. The deep features extracted after six MBRHAB modules are denoted by $F_{M}$, calculated as (3). Both the CBAM and HAG modules maintain the original image dimensions, which enables summations within the MBRHAB module.

#### 2.2.2. Hybrid Attention Group (HAG)

##### Window-Based Multi-Head Self-Attention

This section discusses the core components of the HAG, a window-based MSA mechanism derived from a Swin-Transformer [17]. Existing CNN-based SR reconstruction methods used for remote sensing often suffer from limited convolutional receptive fields and difficulties with establishing global information. The MSA mechanism offers an effective strategy for addressing this issue. In this step, correlations between element positions are utilized to assign weights to each position, thereby adaptively aggregating information from different locations. However, vectorizing 2D images can lead to excessively large vectors, which increases the computational complexity. A window-based MSA mechanism was thus employed to address this issue, with specific implementation steps as follows:

1. The image is divided into uniformly sized windows through partitioning. Each window is then further subdivided into numerous patches of equal size.
2. Within each window, a window-based MSA (W-MSA) step is applied to compute attention metrics among patches.
3. Window shifting is then employed, thereby establishing a shift W-MSA (SW-MSA) to facilitate information exchange between the windows.
4. Patch merging is applied after calculating attention values at specific scales. This involves combining smaller patches into larger ones and establishing a new window scale. Attention calculations are then repeated at the new scale to facilitate global modeling of image features across different hierarchical levels. The formula used for computing self-attention [17] can be expressed as follows:
  (4) $$\mathrm{Attention}\left(Q,K,V\right)=\mathrm{Softmax}\left(\frac{QK^{T}}{\sqrt{d}}+B\right)V,\;Q,K,V\in R^{M^{2}\times d}$$
  where *Q*, *K*, and *V* represent query, key, and value matrices, respectively; $M^{2}$ denotes the number of patches in a window; *d* is the dimension of the *Q* or *K* matrices; and *B* denotes learnable relative positional encoding.

**Figure 2:**
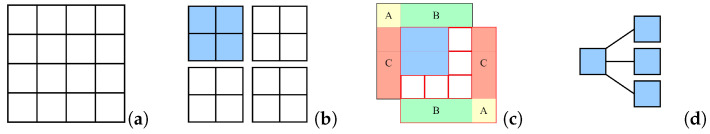
The process of self-attention calculation. (**a**) Patch Partition. (**b**) W-MSA. (**c**) SW-MSA. (**d**) Attention computation.

Figure 2 provides a schematic diagram illustrating the self-attention calculations discussed above. Specifically, Figure 2a shows the feature map after patch partitioning, in which each small square represents an individual patch. Figure 2b demonstrates the calculation of attention among patches within each window using the W-MSA. Figure 2c shows an SW-MSA operation, included to improve information interactions between windows. Finally, Figure 2d depicts attention computations between patches within a window.

##### Structure and Function of the HAG

**Figure 3:**
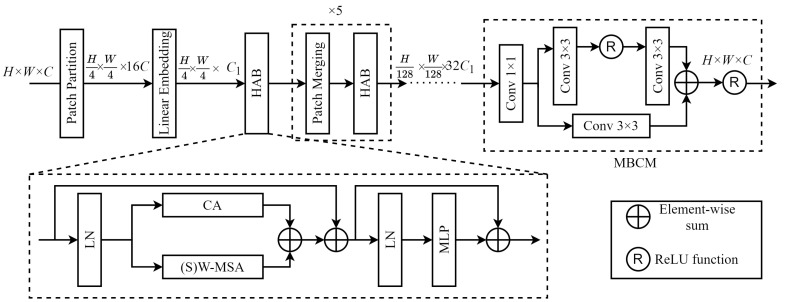
The hybrid attention group (HAG) structure.

Figure 3 shows the overall HAG structure, which primarily consists of six hybrid attention blocks (HABs) and a multi-branch convolution module (MBCM). The structure and function of each sub-module within the HAG can be summarized as follows.

**Figure 4:**
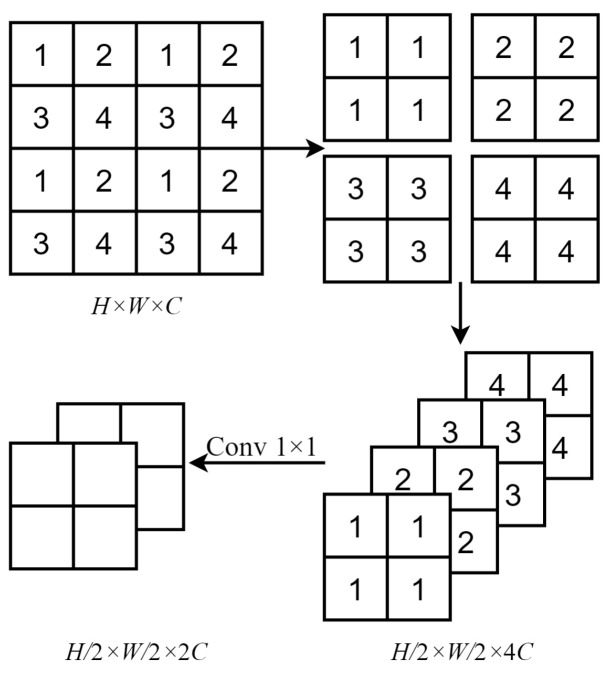
A schematic diagram of the patch merging process.

1. Patch Partitioning:
  Feature maps were first divided into patches of the same size, which were then grouped into the required window size. After shallow feature extraction, the feature map dimensions of H×W×C were converted to (H/4)×(W/4)×16C after patch partitioning. An initial patch size of 4×4 was employed, based on empirical results.
2. Linear Embedding:
  Linear embedding transformed image dimensions after patch partitioning from 16C to a value suitable for MSA (set to $C_{1}$ = 64 in this study).
3. Hybrid Attention Block (HAB):
  Each HAB included W-MSA or SW-MSA (i.e., (S)W-MSA), CA, layer normalization (LN), and a multilayer perceptron (MLP). The parallel configuration of the (S)W-MSA and CA enabled the model to effectively capture long-range dependencies while simultaneously focusing on inter-channel feature correlations, thereby enhancing feature representation capabilities.
  (a) Taking *X* as input and using the HAB, combined with W-MSA, as a feature extraction unit. The output $X_{w}$ can then be described by the following expression:
    (5) $$X_{w1}=f_{W\text{-}MSA}\left(f_{LN}\left(X\right)\right)+f_{CA}\left(f_{LN}\left(X\right)\right)+X$$
    (6) $$X_{w}=f_{MLP}\left(f_{LN}\left(X_{w1}\right)\right)+X_{w1}$$
  (b) Taking $X_{w}$ as input and using the HAB, combined with SW-MSA, as a feature extraction unit. The output $X_{s}$ can then be described by the following expression:
    (7) $$X_{s1}=f_{SW\text{-}MSA}\left(f_{LN}\left(X_{w}\right)\right)+f_{CA}\left(f_{LN}\left(X_{w}\right)\right)+X_{w}$$
    (8) $$X_{s}=f_{MLP}\left(f_{LN}\left(X_{s1}\right)\right)+X_{s1}$$
    where $f_{LN}\left(\cdot \right)$ denotes layer normalization, $f_{\mathrm{MLP}}\left(\cdot \right)$ is a multilayer perceptron, $f_{CA}\left(\cdot \right)$ denotes channel attention, and $f_{W\text{-}MSA}\left(\cdot \right)$ and $f_{SW\text{-}MSA}\left(\cdot \right)$ represent the W-MSA and SW-MSA operations, respectively. It should be noted the HAB itself does not change the size or dimensions of the image. However, after each HAB, the feature map length and width are reduced by half due to patch merging. The number of channels is also doubled and the image size becomes $\left(H/128\right)\times \left(W/128\right)\times 32C_{1}$ due to the HAB step.
4. Patch Merging:
  The purpose of patch merging in the HAG is to construct a hierarchical framework, which allows each HAB to extract image features at various levels, effectively down-sampling the feature maps. This principle involves merging adjacent patches to form newly sized windows after computing attention values within the same scale windows. This process is then repeated in subsequent rounds of attention computation, enabling the acquisition of multi-scale features in remote sensing images. Figure 4 illustrates the patch merging process, in which a feature map of size H×W×C is down-sampled by a factor of two after patch merging. In this process, patches of the same index are merged and then concatenated along the channel dimension. This changes the feature map dimensions to (H/2)×(W/2)×4C. A 1×1 convolution was then used to reduce the number of channels to 2C, resulting in a feature map of size (H/2)×(W/2)×2C.
5. Multi-branch Convolutional Module (MBCM):
  A novel multi-branch convolutional module (MBCM) was included in the model to increase the size of the convolutional receptive field and integrate deep feature information extracted by the HAB. This enhancement aimed to improve feature representations for global information. The module simultaneously restored the feature map size to its original value of H×W×C. The structure of the MBCM, which involved stacking convolutions of the same size but different depths across various branches, is depicted in the dashed box of the MBCM section in Figure 2. MBCM is first a 1×1 convolution that is used to recover the channel dimension of the feature. Then the upper branch in the MBCM consisted of two consecutive 3×3 convolutional layers with a ReLU activation function between them. The lower branch consisted of a single 3×3 convolutional layer. Elements from both branches were added together and passed through a ReLU activation function before outputting the final feature information. The design of these two branches was intended to enrich the expression of feature information by using convolutional receptive fields of different sizes. Specifically, the upper branch employed two 3×3 convolution layers instead of a single 5×5 layer. This choice not only increased model depth and reduced the number of parameters, it also enhanced the model’s nonlinear expressive capacity by using nonlinear activation functions in the convolution layers.

#### 2.2.3. Convolutional Block Attention Module (CBAM)

**Figure 5:**
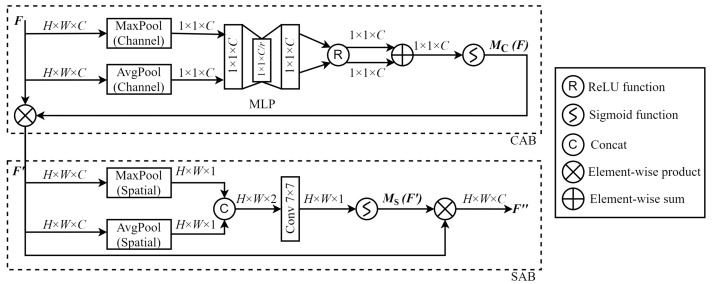
The convolutional block attention module (CBAM) structure.

The use of a single global feature extraction process can cause the model to pay insufficient attention to locally important image features, particularly small yet critical details in remote sensing images (e.g., buildings and traffic signs). As such, this study expands the use of MSA mechanism for global long-range dependency modeling by integrating a CBAM [21] in parallel. This integration enhanced the model’s ability to incorporate local information from various dimensions of remote sensing images, significantly improving its ability to detect and interpret these subtle features. The CBAM incorporates both CA and SA mechanism, as shown in Figure 5. Initially, the CA reorganizes the channel dimensions to refine the feature set. The SA is subsequently used to compute features, an approach designed to reduce the computational burden and complexity. The CBAM structure and function are elaborated further below.

1. Channel Attention Block (CAB):
  The CAB processes channel dimensions and compresses feature maps in the spatial dimension, such that features in different channels are assigned various importance weights. This process can be described by the following steps.
  (a) A feature map *F* of size H×W×C is processed through both channel max pooling and average pooling to obtain two feature maps of size 1×1×C.
  (b) These two feature maps are processed separately using a MLP. After processing, the maps are activated by a ReLU function and then combined by addition, resulting in a feature map of dimensions 1×1×C.
  (c) $M_{C}\left(F\right)$ is the result of CA obtained using a sigmoid activation function. This result is then combined with the input feature *F* to produce $F^{\prime }$, while the feature size remains unchanged H×W×C.
2. Spatial Attention Block (SAB):
  After acquiring importance differences between various channels, the feature map is then compressed in the channel dimension by the SAB, precisely extracting features at each spatial location. This process can be described by the following steps.
  (a) Spatial average pooling and maximum pooling are performed in the channel dimension for each feature map $F^{\prime }$ that has passed through CAB, producing two H×W×1 feature maps.
  (b) These maps are then concatenated to form a new feature map of size H×W×2, and the combined map is then processed using a 7×7 convolution to produce a map of dimensions H×W×1.
  (c) $M_{S}\left(F^{\prime }\right)$ is the result of SA obtained through the sigmoid activation function. This result is multiplied by the input feature $F^{\prime }$ to obtain $F^{\prime \prime }$, and the feature sizes of $F^{\prime }$ and $F^{\prime \prime }$ are still H×W×C.

### 2.3. Image Reconstruction

The image reconstruction component utilizes sub-pixel and 3×3 convolutional layers to compose images, which are recovered as three-channel HR images. This process can be expressed as

(9) $$I_{\mathrm{HR}}=C_{3\times 3}\left(SubPixelCov\left(F_{0}+F_{M}\right)\right)$$

where $F_{0}$ represents shallow features, $F_{M}$ denotes deep features, SubPixelCov(·) signifies a sub-pixel convolution, $C_{3\times 3}\left(\cdot \right)$ represents a 3×3 convolution, and $I_{\mathrm{HR}}$ denotes an HR image produced after model reconstruction.

## 3. Experiments

### 3.1. Datasets for Experiments

This study utilized publicly available remote sensing datasets, including NWPU-RESISC45 [22], PatternNet [23] and UCMerced-LandUse [24].

The NWPU-RESISC45 dataset [22] comprises 31,500 images across 45 scene categories, including airport, basketball court, beach, bridge, farmland, commercial area, forest, freeway, lake, meadow, etc. Each category contains 700 images and has a size of 256 × 256 pixels in the red–green–blue (RGB) color space. The spatial resolution of this dataset varies from about 0.2 m to 30 m per pixel for most of the scene categories, except for the island, lake, mountain, and snow berg categories, which have lower spatial resolutions. This dataset was extracted from Google Earth (Google Inc.), and it maps more than 100 countries and regions via the superimposition of images obtained from satellite imagery, aerial photography, and geographic information system (GIS) onto a 3-D globe.

The PatternNet dataset [23] consists of 30,400 images across 38 scene categories, including airplane, baseball field, beach, bridge, cemetery, chaparral, closed road, crosswalk, etc. Each category contains 800 images, with a size of 256 × 256 pixels in the RGB color space. This dataset contains images with varying resolutions. The highest spatial resolution is around 0.062 m, and the lowest spatial resolution is around 4.693 m. The dataset was collected from Google Earth imagery or via the Google Map API for US cities.

The UCMerced-LandUse dataset [24] includes 2100 images across 21 scene categories, including agricultural, airplane, baseball diamond, beach, buildings, chaparral, dense residential, forest, freeway, etc. Each category contains 100 images. The image resolution is 256 × 256 pixels in the RGB color space, and the spatial resolution is around 0.3 m per pixel. The dataset was extracted from aerial orthoimagery downloaded from the United States Geological Survey (USGS) National Map of some US regions.

### 3.2. Experimental Setup

Model effectiveness was validated using cross-dataset training, in which training and testing samples were selected from different datasets. Specifically, the model was trained and validated on NWPU-RESISC45 and PatternNet and tested on UCMerced-LandUse. The training set consisted of 30 images randomly selected from each category of NWPU-RESISC45 and PatternNet, totaling 2490 images. The validation set included two additional images randomly selected from each category of the training database, totaling 166 images. The test set was comprised of five images randomly selected from each category of the UCMerced-LandUse dataset, totaling 105 images.

LR images required for the experiments were derived from HR images using bicubic downsampling. The reasons we chose the bicubic downsampling method are as follows. Bicubic downsampling [25] and Gaussian blur downsampling [26] are usually used to obtain LR images for SR reconstruction. Among them, bicubic downsampling calculates the value of each pixel in the LR image through power exponential weighted sum of the values of the surrounding pixels. Gaussian blur downsampling first applies the Gaussian blur function to the original image to reduce the high-frequency noise, and then performs downsampling. Gaussian downsampling is more commonly used in situations with denoising requirements. Bicubic downsampling can obtain smoother downsampling results, and its performance is closer to that of actual LR image acquisition devices.

All experiments were conducted on a PC with an Intel Xeon(R) Gold 6271C 2.60Hz CPU (Intel, Santa Clara, CA, USA), an NVIDIA GeForce RTX 4090(24GB) GPU × 2 (Nvidia, Santa Clara, CA, USA), 128 GB of RAM, and Python 3.10. Data augmentation techniques such as horizontal flipping and rotation were employed during the training process. Absolute error loss was employed as the loss function and the model was trained with a batch size of 16, for a total of 50,000 iterations, with an initial learning rate set to 2 × $10^{-4}$ and halved every 10,000 iterations. All of the metric results for validation or test are the average of the SR reconstruction results for all of the samples in the validation set or test set. That is, for the validation, the number of iterations is 166, and for the test, the number of iterations is 105.

Model performance was evaluated using the peak signal-to-noise ratio (PSNR) and structural similarity index (SSIM) [27] as metrics. Higher PSNR and SSIM values indicated smaller discrepancies between reconstructed and original images, signifying better image quality.

### 3.3. Experimental Contents

We conducted a total of four sets of experiments: ablation experiments, model feasibility assessment, comparative experiments, and test experiments for the different categories.

An ablation experiment was conducted to determine the impact of the CBAM (shown in Figure 1) and the MBCM sub-module (shown in Figure 3) on the SR reconstruction of remote sensing images. The basic model, excluding the CBAM and MBCM modules, served as a baseline. The MBCM sub-module was added first, followed by the CBAM. All models were configured with identical parameter settings and followed the same training strategy.

Model feasibility assessment was conducted to demonstrate the parameter tuning process and convergence analysis of the model through its performance on the validation set during training. In this experiment, the learning rate was halved every 5000 iterations during 50,000 training iterations, and the image SR reconstruction performance was tested on the validation set. These values were then used to evaluate the feasibility of our model.

In the comparative experiment, a series of experiments were conducted to compare the model developed in this study with SR image reconstruction methods based on bicubic interpolation, CNNs, GANs, and attention mechanisms. These included Bicubic interpolation [25], SRCNN [7], IRN [9], ESRGAN [11], Real-ESARGAN [12], and DSSR [19]. The results were analyzed in terms of both objective evaluation metrics and subjective visualization.

In order to analyze the SR reconstruction performance of the proposed model in different remote sensing scenarios in more detail, we conducted tests on 21 different scene categories in the UCMerced-LandUse database. The test results for all of the categories are the average metrics of the five sample images in each category.

## 4. Results

### 4.1. Objective Evaluation Metrics

#### 4.1.1. Ablation Experiments

PSNR and SSIM values for each model used in the ablation experiment are shown in Table 1. Compared with the baseline model, the addition of an MBCM submodule improved PSNR and SSIM values by 0.18 and 0.030, respectively, for an amplification factor of 2. In addition, PSNR and SSIM values improved by 0.34 and 0.0047, respectively, for an amplification factor of 3 and by 0.48 and 0.0051, respectively, for an amplification factor of 4. These improvements validated the effectiveness of the MBCM for enhancing feature expressions with global information, by effectively integrating deep feature information.

The impact of the CBAM on model performance was also verified. As seen in Table 1, integration of the CBAM module produced noticeable improvements at multiple amplification factors. For example, PSNR and SSIM values increased by 0.53 and 0.0082, respectively, for an amplification factor of 2, 0.91 and 0.0105, respectively, for a factor of 3, and 1.10 and 0.0121, respectively, for a factor of 4. These results demonstrate that model capacity for deep feature extraction can be effectively improved by long-range dependency modeling using windowed multi-head self-attention, combined with the local extraction capabilities of the CBAM module.

#### 4.1.2. Model Feasibility Assessment

**Figure 6:**
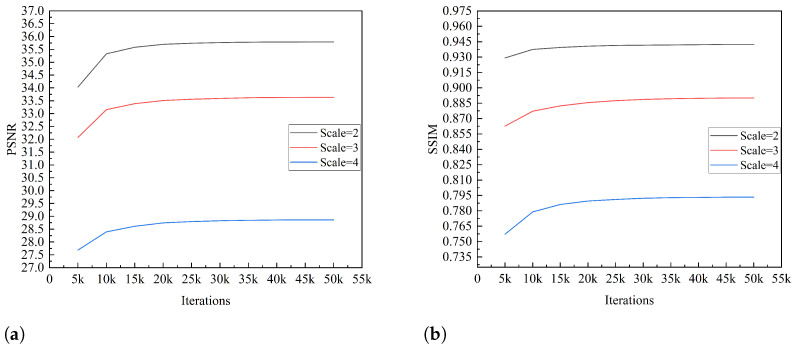
Metric curves on the validation set. (**a**) PSNR-Iterations curves. (**b**) SSIM-Iterations curves.

Figure 6a,b show the curves of the PSNR and SSIM performances, respectively, for our model on the validation set over 50,000 training iterations when the amplification factors are 2, 3, and 4. The metric curve indicates that when the number of training iterations is within 10,000, the super-resolution reconstruction performance of the model improves rapidly. When the number of training iterations reaches 10,000–35,000, the PSNR and SSIM of the model’s super-resolution reconstruction gradually stabilize, and the model’s performance slowly improves. When the number of training iterations exceeds 35,000, the PSNR and SSIM of the model reach saturation, and the performance of the model converges.

#### 4.1.3. Comparative Experiments

Table 2 provides PSNR and SSIM results for each model applied to the test set at magnification factors of 2, 3, and 4, with bicubic interpolation producing the lowest values, followed by the SRCNN method based on a convolutional neural network. In contrast, the ESRGAN and Real-ESARGAN, which are GAN-based image SR reconstruction algorithms, demonstrated superior performance. Specifically, the IRN model achieved more efficient SR reconstruction results than GAN-based methods by iterating the residual network structure. The DSSR model included a widely characterized attention block that enhanced network representation and produced sub-optimal PSNR and SSIM values. The proposed model, which combined multiple attention mechanisms to process global and local features in parallel, improved the quality and clarity of the reconstructed images. As shown in Table 2, the proposed model achieved the best SR reconstruction results, with PSNR and SSIM values of 35.65 and 0.9388 at a magnification factor of 2, respectively, 33.38 and 0.8860 at a factor of 3, and 28.40 and 0.7850 at a factor of 4, thereby validating the effectiveness of the proposed technique.

#### 4.1.4. Test Experiments for Different Categories

Table 3 presents the PSNR and SSIM results for 21 categories of remote sensing images in the UCMerced-LandUse database reconstructed using this model at magnifications of 2, 3, and 4. When the amplification factor is 2, the range of the PSNR value is 31.28 dB to 41.22 dB (data with two decimal places retained) and the range of the SSIM value is 0.8682 to 0.9784. When the amplification factor is 3, the range of the PSNR value is from 28.90 dB to 39.23 dB and the range of the SSIM value is 0.7825 to 0.9569. At a magnification of 4 times, the range of the PSNR value is 23.57 dB to 33.40 dB and the range of SSIM value is 0.6322 to 0.8919.

At all three different magnifications, the PSNR and SSIM of the SR reconstruction for the beach category have the best performances, while they have the lowest performances for the medium density residential category. In addition, the model performs well in remote sensing image scenes containing categories such as the baseball diamond, golf course, and runway, but it performs weakly in image environments containing categories such as the parking lot, harbor, and overpass. This indicates that the model has the best reconstruction effect on images with clear boundaries and smooth textures such as those containing beaches and sea areas, and it has a better reconstruction effect on remote sensing images with regular lines and relatively uniform textures such as those containing baseball fields and runways. The reconstruction performance of the images containing medium density residential areas indicates that the model proposed in this approach still has difficulty in dealing with dense structures and complex textures.

### 4.2. Subjective Visualization

**Figure 7:**
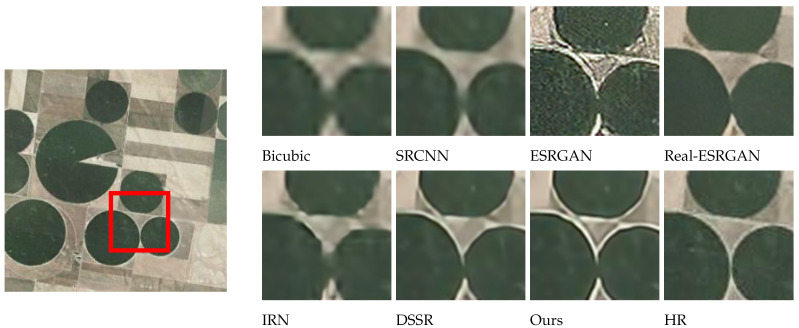
A comparison of reconstruction effects in the wheat field category (validation set).

**Figure 8:**
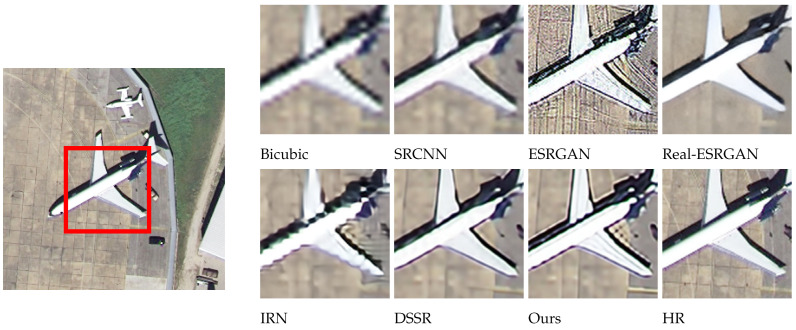
A comparison of reconstruction effects in the air-plane category.

**Figure 9:**
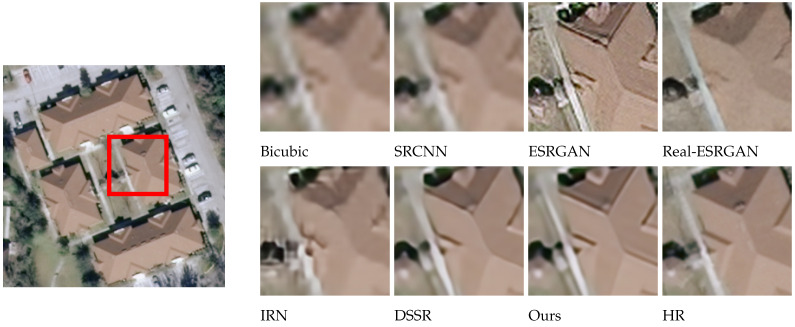
A comparison of reconstruction effects in the dense residential category.

**Figure 10:**
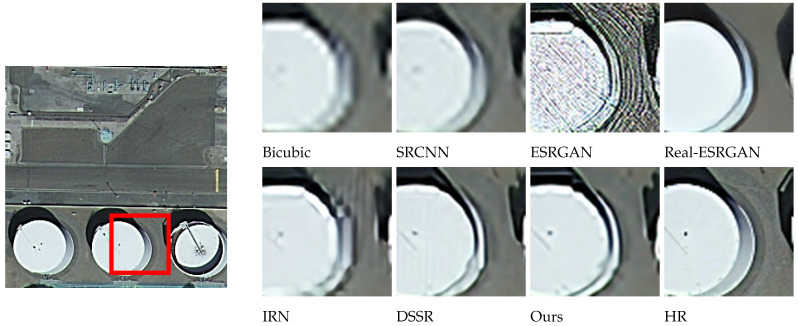
A comparison of reconstruction effects in the storage tanks category.

The effects of different models on SR reconstruction performance were also analyzed qualitatively, using images from four different categories: wheat field, airplane, dense residential, and storage tanks. These samples were randomly selected and used to compare reconstruction effects at a magnification factor of 4. As seen in Figure 7, Figure 8, Figure 9 and Figure 10, images reconstructed using the Bicubic interpolation are blurry. The SRCNN algorithm achieved better results, though some blurring was still evident. Details in the ESRGAN image were sparse, resulting in chaotic image textures and excessive noise in the reconstructed samples. In contrast, the Real-ESRGAN reconstructions were overly smooth and lacked detail, making them less realistic. The IRN reconstructions included multiple artifacts, which obstructed spatial information. The DSSR algorithm was not very consistent across samples, as vertical artifacts were evident in some cases, such as the storage tanks scene shown in Figure 10. However, the model proposed in this study produced remote sensing images with superior object contours, texture detail, and image clarity, more closely resembling the real HR images.

## 5. Discussion

We obtained corresponding experimental results and analysis results through a series of experiments on SR reconstruction of remote sensing images. In this section, we discuss the details of the data and the principle analysis of each set of experiments.

In the ablation experiments, it was observed that the addition of MBCM and CBAM submodules improved the PSNR and SSIM values of the reconstructed images. This is because the MBCM submodule can effectively integrate the global features extracted utilizing window MSA and CA in order to reasonably improve the attention to dense details such as the building density and road distribution in remote sensing images, thereby increasing the effectiveness and robustness of the model. In addition, the CBAM submodule can enable the model to extract important edge information such as coastlines and roads, as well as texture information such as forests and grassland, to provide supplementing important local information. Furthermore, it should be noted that compared to the PSNR, the improvement of the SSIM is in the small thousandth range (such as 0.0047), which may be because the SSIM value ranges from -1 (completely different) to 1 (exactly the same). In addition, this could be because the SSIM focuses more on structural differences rather than subtle noise, so its value is less sensitive to some small changes when approaching 1.

Regarding the model feasibility assessment experiment, the SPNR and SSIM curves of the validation set reflect the convergence trend of the model during the training process.It can be observed from Figure 6 that the PSNR and SSIM of the SR reconstructed images for the validation set are slightly higher than those for the test set. This is because both the training and validation sets are from the NWPU-RESISC45 and PatternNet datasets, while the test set is from the UCMerced-LandUse dataset. These conclusions demonstrate the effectiveness of dataset cross validation.

Regarding the comparative experiments, Table 2 confirms that our model achieved the optimal PSNR and SSIM values on the testing set when the amplification factors were 2, 3, and 4. As can be seen from Figure 7, Figure 8, Figure 9 and Figure 10, the image reconstructed using the Bicubic interpolation algorithm is the most blurry, corresponding to the lowest PSNR and SSIM values. The edge of the image reconstructed using the SRCNN method based on convolutional neural networks is clearer than based on the Bicubic interpolation algorithm, but the rest of the image is still blurry. Moreover, for dense building scenes such as the parking lot and the harbor categories, its edge reconstruction performance is poor, with the second worst PSNR and SSIM values. The ESRGAN and real ESARGAN exhibit better performances than the previous two. The ESRGAN algorithm has the best edge reconstruction effect, but it also generates more texture noise. The IRN algorithm can attain higher PSNR and SSIM values compared to the GAN-based networks, but its subjective effect on SR reconstruction is not good, which suggests that evaluating image reconstruction algorithms solely based on existing objective indicators has certain limitations. The DSSR model enhances the network’s representation ability by designing feature attention blocks, achieving suboptimal algorithm performance. However, the algorithm still suffers from the loss of edge details in terms of subjective effects due to a slight lack of detail feature extraction in the DSSR model. Our SR reconstruction model achieved the best reconstruction effect, with clear edges and rich texture details in terms of the subjective effects. This is because our model combines global and local image features through multiple attention mechanisms, effectively reconstructing clear images using global semantic information while preserving important local information such as edge contours and texture details. However, it should be noted that in certain scenes with relatively single image content and a large ground size, such as wheat fields and airplanes in Figure 7 and Figure 8, due to the algorithm repeatedly focusing on edge features in the attention mechanism, the edge features are excessively reinforced, resulting in our model experiencing over-sharpening. Next, we investigate the automatic adjustment of attention weights for different content sizes with the aim of finding a solution to this phenomenon.

Through the test experiments for the different categories, it was found that our model has the best SR reconstruction performance for the beach category, followed by the baseball diamond, golf course, and runway categories, while its performance for the medium density residential category is the lowest. This is because the proposed model has the best reconstruction effect on remote sensing images containing clear boundaries and smooth textures, followed by images containing regular lines and relatively uniform textures, and it has the worst reconstruction effect on images containing dense structures and complex textures. However, as can be seen from Figure 9, our model has the best reconstruction performance for the dense residential categories compared to the other algorithms. This indicates that our model has made breakthroughs in image scenes containing dense structures and complex textures, but further improvements of the algorithm still need to be studied for such scenes.

The image SR reconstructed using our model has practical applications in remote sensing, image segmentation, and object detection. Additional studies should be conducted on these joint algorithms.

## 6. Conclusions

This work presents an SR reconstruction model for remote sensing images, which combine multiple attention mechanisms. The model features a novel MBRHAB, which combines a HAG in parallel with a CBAM. The resulting windowed MSA mechanism in the HAG extracts global image information from images by modeling long-range dependencies. Simultaneously, the design of the MACM effectively integrates global image information. The CA and SA in the CBAM also provided important textural and edge detail features.

We used a cross-dataset approach to validate the effectiveness and generalization capabilities of the proposed model. The two training datasets are NWPU-RESISC45 and PatternNet, and the test dataset is UCMerced-LandUse. Experimental results produced a PSNR of 35.65 dB and an SSIM of 0.9388 at ×2 magnification, a PSNR of 33.38 dB and an SSIM of 0.8860 at ×3 magnification, and a PSNR of 28.40 dB and an SSIM of 0.7850 at ×4 magnification. The experimental results confirm that the proposed remote sensing image SR reconstruction model outperforms existing algorithms in both objective evaluation metrics and subjective visualization.

Future work will focus on reducing the number of parameters, simplifying the model structure, and research on joint algorithms for SR reconstruction, object segmentation, and detection.

## Figures and Tables

**Table 1:** PSNR and SSIM values after the addition of MBCM and CBAM modules.

Baseline	MBCM	CBAM	Scale	PSNR/dB	SSIM
1	0	0	×2	35.12	0.9306
1	1	0	×2	35.30	0.9336
1	1	1	×2	35.65	0.9388
1	0	0	×3	32.47	0.8755
1	1	0	×3	32.81	0.8802
1	1	1	×3	33.38	0.8860
1	0	0	×4	27.30	0.7729
1	1	0	×4	27.78	0.7780
1	1	1	×4	28.40	0.7850

**Table 2:** PSNR and SSIM values for each model applied to the test set.

Model	Scale ×2		Scale ×3		Scale ×4	
	PSNR/dB	SSIM	PSNR/dB	SSIM	PSNR/dB	SSIM
Bicubic	33.26	0.8677	32.23	0.8416	27.05	0.7708
SRCNN	34.50	0.9045	32.46	0.8578	27.47	0.7735
ESRGAN	34.83	0.9158	32.54	0.8627	27.58	0.7744
Real-ESRGAN	34.92	0.9217	32.70	0.8694	27.77	0.7772
IRN	35.21	0.9302	32.89	0.8742	27.93	0.7789
DSSR	35.48	0.9341	33.11	0.8809	28.12	0.7821
**Ours**	**35.65**	**0.9388**	**33.38**	**0.8860**	**28.40**	**0.7850**

**Table 3:** PSNR/SSIM values for each category on Ucmerced Landuse dataset.

Category	Scale ×2		Scale ×3		Scale ×4	
	PSNR/dB	SSIM	PSNR/dB	SSIM	PSNR/dB	SSIM
agricultural	36.9389	0.8897	35.1506	0.8141	30.1999	0.6503
airplane	36.6847	0.9419	34.3808	0.8925	28.6323	0.8104
baseball-diamond	41.1253	0.9638	39.0854	0.9376	33.2661	0.8575
beach	41.2185	0.9784	39.2264	0.9569	33.4048	0.8919
buildings	35.4007	0.9533	32.8697	0.9091	28.3992	0.8260
chaparral	33.4573	0.9252	31.2497	0.8584	26.5914	0.7333
dense-residential	37.7685	0.9679	34.4910	0.9364	31.5699	0.8564
forest	33.2402	0.9110	31.1615	0.8005	26.0286	0.6979
freeway	36.7079	0.9393	33.9108	0.8806	28.9242	0.8204
golf-course	38.7320	0.9494	37.8070	0.9226	32.1114	0.8522
harbor	32.6609	0.9746	30.3614	0.9357	24.5044	0.8249
intersection	33.5985	0.9410	31.3754	0.8700	25.8833	0.7562
medium-density-residential	31.2835	0.8682	28.9026	0.7825	23.5688	0.6322
mobile-home-park	36.8960	0.9759	34.0592	0.9510	29.5797	0.8603
overpass	32.8210	0.9316	30.7549	0.8670	24.6546	0.7451
parking-lot	32.1953	0.9514	29.5809	0.8991	24.4980	0.7826
river	35.4638	0.9332	33.1560	0.8686	28.4227	0.7588
runway	38.5161	0.9453	37.0427	0.9063	32.6700	0.8232
sparse-residential	33.5898	0.8915	31.3646	0.8526	26.8750	0.7045
storage-tanks	35.1514	0.9444	32.4735	0.8954	28.3216	0.8199
tennis-courts	35.2662	0.9386	32.6238	0.8695	28.3791	0.7821
Average	35.6532	0.9388	33.3823	0.8860	28.4041	0.7850

## Data Availability

The data presented in this study are derived from public domain resources. The data are available at https://gcheng-nwpu.github.io/#Datasets for the NWPU-RESISC45 datset, https://sites.google.com/view/zhouwx/dataset for the PatternNet dataset, and http://weegee.vision.ucmerced.edu/datasets/landuse.html for the UC Merced Land Use dateset.
